# Thickness Influences on Structural and Optical Properties of Thermally Annealed (GaIn)_2_O_3_ Films

**DOI:** 10.3390/nano15181385

**Published:** 2025-09-09

**Authors:** Shiyang Zhang, Fabi Zhang, Tangyou Sun, Zanhui Chen, Xingpeng Liu, Haiou Li, Shifeng Xie, Wanli Yang, Yue Li

**Affiliations:** 1 Key Laboratory of Microelectronic Devices and Integrated Circuits, Guilin University of Electronic Technology, Guilin 541004, China; zsy2023@mails.guet.edu.cn (S.Z.); suntangyou@guet.edu.cn (T.S.); zhchen@guet.edu.cn (Z.C.); tadyliu@guet.edu.cn (X.L.); lihaiou@guet.edu.cn (H.L.); 2 Guangxi Key Laboratory of Precision Navigation Technology and Application, Guilin University of Electronic Technology, Guilin 541004, China; 3 The 34th Research Institute of China Electronics Technology Group Corporation, Guilin 541004, China; xieshifeng@cetc.com.cn (S.X.); yangwanli1@cetc.com.cn (W.Y.); 15507738324@wo.cn (Y.L.)

**Keywords:** (GaIn)_2_O_3_ film, pulsed laser deposition, thickness, ion bombardment

## Abstract

This work explores the relationship between the thickness and the structural, morphological, and optical features of thermally annealed (GaIn)_2_O_3_ thin films grown by pulsed laser deposition at room temperature. The thickness of the (GaIn)_2_O_3_ films varied from 20 to 391 nm with an increase in deposition time. The film with a thickness of about 105 nm showed largest grain size as well as the strongest XRD peak intensity, as measured by atomic force microscopy and X-ray diffraction. The studies on the optical properties show that the bandgap value decreased from 5.14 to 4.55 eV with the change in the film thickness from 20 to 391 nm. The film thickness had a significant impact on the structure, morphology, and optical properties of (GaIn)_2_O_3_, and the PLD growth mode notably influenced the film quality. The results suggest that optimizing the film thickness is essential for improving the film quality and achieving the target bandgap.

## 1. Introduction

Group-III sesquioxides have interesting physical properties such as a wide bandgap range (from about 8.8 eV for Al_2_O_3_ to 2.9 eV for In_2_O_3_), physical and chemical stabilities, and a high breakdown voltage, rendering these materials suited for high-power applications or solar-blind photo detectors [1]. Doping of group-III sesquioxides to form alloyed oxides offers a promising approach to tune the electronic structure, particularly the band gap, and to tailor the desired physical properties for specific applications. (GaIn)_2_O_3_ is an interesting material system among sesquioxides. Indium, acting as a surfactant, can activate a more favorable growth mechanism through surface wetting, effectively improving the crystal quality of (GaIn)_2_O_3_ [2]. The bandgap value of (GaIn)_2_O_3_ can be varied from 4.9 eV to 3.4 eV, and it is more transparent than other amorphous oxide semiconductors [3], particularly in the blue and green spectral regions. It thus holds application potential in ultraviolet photodetectors, transparent electronic devices, and resistive memory.

Numerous synthesis approaches have been reported for this ternary system, including molecular beam epitaxy [4], metal organic chemical vapor deposition [5], sputtering [3,6,7], atomic layer deposition [8,9], the sol–gel method [10] and pulsed laser deposition (PLD) [11]. PLD stands out among deposition techniques due to its ability to grow films with compositions very similar to the original target [12,13]. We have grown bandgap-tunable (GaIn)_2_O_3_ films by PLD. However, phase separation occurred on (GaIn)_2_O_3_ films deposited at high temperature with a low indium content [14]. We have found that by thermally annealing the film deposited at RT by PLD, crystalline (GaIn)_2_O_3_ film without phase separation can be obtained [15]. We have also optimized the annealing parameters such as temperature and ambient conditions [16]. Besides the process parameters, the film thickness is another factor which should also be taken into account because it has a considerable effect on the structural, electrical, and optical properties of a film [17]. For example, the dielectric constant and remnant polarization of lead zirconate titanate thin films decrease with decreasing film thickness, while the coercive field increases [18]. The crystal quality of ZnO: Al(AZO) film was improved with the increase in thickness, while the resistivity decreased [17]. An et al. have prepared Ga_2_O_3_ films by radio frequency magnetron sputtering, and they found that the film thickness influences the photoelectric properties [19]. For (GaIn)_2_O_3_ films with tunable bandgaps, the thickness significantly affects both crystal quality and the attainment of the targeted bandgap. Understanding these thickness effects is crucial for device optimization and can guide the selection of an optimal thickness that balances performance, surface morphology, and fabrication efficiency. In this work, the influence of the film thickness on the structural and optical properties of thermally annealed (GaIn)_2_O_3_ films grown by PLD are discussed.

## 2. Materials and Methods

The (GaIn)_2_O_3_ films for annealing were prepared by pulsed laser deposition on (0001) sapphire substrates at room temperature (RT). Prior to deposition, the sapphire substrates were chemically etched in a hot H_3_PO_4_:H_2_SO_4_ (1:3) solution and then rinsed in deionized water before loading into the growth chamber. The (GaIn)_2_O_3_ (99.99%) target was mounted to a holder which was rotated during the growth. The In content In/(Ga + In) (atomic ratio) in the (GaIn)_2_O_3_:Eu target was about 0.23. The laser energy used for deposition was 225 mJ, with a repetition of 1 Hz. The deposition times were 10 min, 30 min, 60 min, 120 min, and 180 min. Post-annealing was carried out with an electrical furnace in air for one hour. The annealing temperature was set at 900 °C.

After annealing, the film thickness was measured by a surface step profile analyzer. The structural characteristics of the films were investigated by X-ray diffraction (XRD) on a PANalytical X’Pert PRO system using CuKα, 40 kV, and a 30 mA emission line at room temperature. The elemental composition was measured by energy dispersive spectroscopy (EDS). Surface morphologies were obtained by an atomic force microscope (AFM) in the contact mode using a Digital instruments Nanoscope, Veeco, MMAFMLN-AM. Optical transmission spectra were measured with a spectrophotometer. Raman and photoluminescence spectroscopy were performed using a 488 nm excitation laser at room temperature.

## 3. Results and Discussion

**Figure 1:**
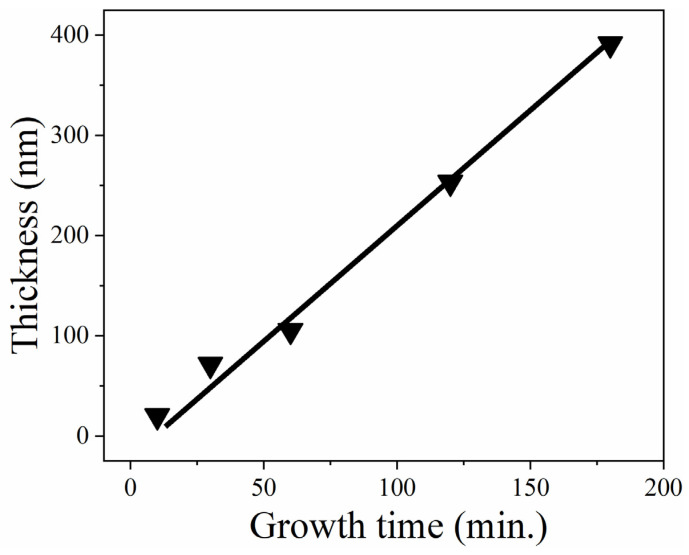
Dependence of thickness on growth time of (GaIn)_2_O_3_ films produced using PLD.

The dependence of the thickness on the growth time of the (GaIn)_2_O_3_ films is shown in Figure 1. The measured thickness of the films deposited for 10, 30, 60, and 180 min were about 20, 71, 105, 253, and 391 nm, respectively. The thickness of the films increased lineally with the deposition time, as shown in Figure 1.

**Figure 2:**
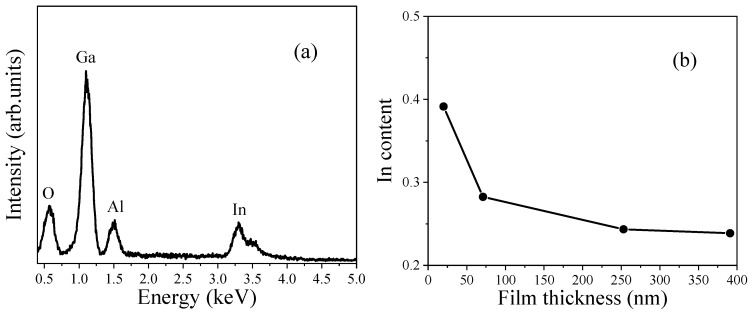
(**a**) Typical EDS spectrum of (GaIn)_2_O_3_ film; (**b**) variation in indium content with film thickness.

In order to clarify the elemental composition of the prepared films, EDS analysis was conducted. Figure 2a shows a typical EDS spectrum of the (Ga_0.24_In_0.76_)_2_O_3_ films. Elements of O, Al, Ga, and In were detected. The Al element was from the sapphire substrate. The presence of Ga and In indicated that the (Ga_0.24_In_0.76_)_2_O_3_ film had formed on the substrate. The obtained indium content as a function of the film thickness is plotted in Figure 2b. The indium contents (In/(Ga + In), atomic ratio) of the (GaIn)_2_O_3_ films with thicknesses of 20, 71, 253, and 391 nm were 0.39, 0.28, 0.24, and 0.24, respectively. The indium content decreased with the increasing in the film thickness for films thinner than 253 nm, and the indium content became steady for thicker films, which was close to that of the target. Similarly, Sakai et al. also observed a certain correlation between film thickness and elemental composition. The uniformity of the thickness distribution is influenced by deposition parameters, which can affect the elemental ratio by altering the diffusion paths of the species [20]. This is closely related to the growth mechanism of PLD. Due to the lattice mismatch between (GaIn)_2_O_3_ and Al_2_O_3_, the initial growth typically follows an island growth mode: atoms first land on the substrate surface, then migrate and diffuse until they reach energetically favorable sites. As the film thickness increases, these islands gradually grow larger and eventually coalesce into a continuous film. The growth mode then transitions to a layer-by-layer process, leading to a continuous improvement in crystalline quality [21,22]. The bonding energies of the various elements in a multi-component target can affect their ablation efficiency; elements with lower bonding energies are more prone to evaporation or ablation under laser irradiation [23]. For example, the In–O bond has a relatively low bonding energy, making indium-rich regions more likely to evaporate during the initial stages of deposition [24]. As the deposition process proceeds, the film thickness increases, bulk-phase deposition becomes dominant, and the target surface reaches a relatively stable ablation state, thereby causing the film composition to gradually approach the intrinsic stoichiometry of the target material.

**Figure 3:**
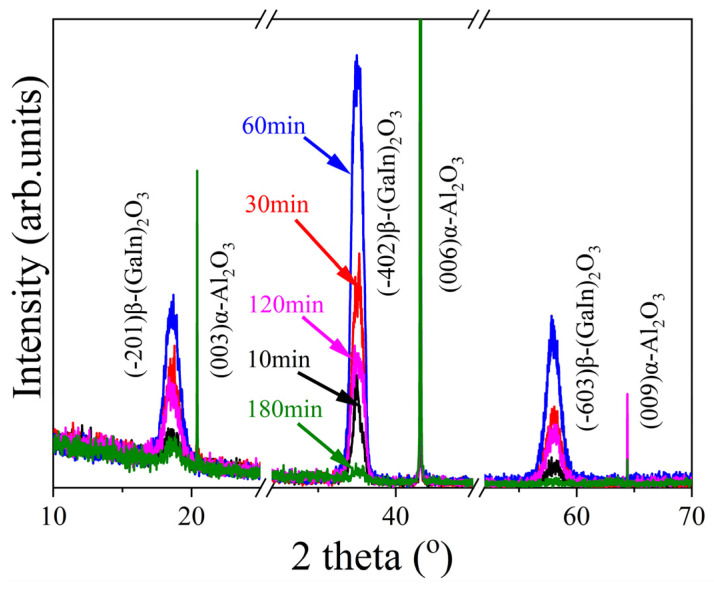
XRD of patterns of (GaIn)_2_O_3_ films as a function of thickness.

X-ray diffraction (XRD) testing was used to investigate the structural properties of the thin films, and the results are shown in Figure 3. The peaks located at 2θ values of 20.4°, 41.6°, and 64.4° come from the sapphire substrate. The diffraction peaks located at 2θ values of about 18.6°, 37.5°, and 58.0° were assigned as the (-201), (-402), and (-603) planes of monoclinic β-(GaIn)_2_O_3_. Those peaks shifted towards lower 2θ angles compared with those of β-Ga_2_O_3_ (JCPDS, PDF No. 43043-1012), indicating the incorporation of indium atoms into the β-Ga_2_O_3_ lattices. It is noticeable that the XRD peak intensity of the (GaIn)_2_O_3_ films varied with the film thickness. The (GaIn)_2_O_3_ film with a thickness of about 105 nm showed the strongest XRD peak intensity. For films thinner than 105 nm, the intensity increased with the film thickness, while for thicker films, the intensity decreased. The full width at half maximum (FWHM) of the 2θ value is related to the grain size of the film. A larger FWHM indicates smaller grain size, more defects, and greater microstrain. The FWHM values of the (-402)β-(GaIn)_2_O_3_ diffraction peak for films with thicknesses of 20 nm, 71 nm, 105 nm, 253 nm, and 391 nm were 0.56, 0.81, 0.89, 1.00, and 1.89, respectively.

To determine the grain size of the samples, we used the classical Scherrer equation [25], which is given by:

(1) $$\begin{array}{c} D=\frac{k\lambda }{\beta cos\theta } \end{array}$$

where *k* is the Scherrer constant, typically taken as 0.9, *λ* is the wavelength of the incident X-ray (0.154 nm), *β* is the FWHM of the diffraction peak due to grain size, measured in radians, and *θ* is the Bragg diffraction angle, also in radians. By calculating the grain size from the XRD diffraction peak of the (-402) plane, the films with thicknesses of 20 nm, 71 nm, 105 nm, 253 nm, and 391 nm had grain sizes of 14.98 nm, 10.36 nm, 9.42 nm, 8.39 nm, and 4.44 nm. From these results, it can be observed that the thinnest films had the largest grain sizes, which is related to the island-like growth mode at the initial stage of PLD growth. As the thickness increased, the grain size gradually decreased.

Using Bragg’s law, we can obtain the experimental interplanar spacing $d_{esp}$ of the films and the standard interplanar spacing $d_{0}$ of (-402) β-Ga_2_O_3_:

(2) $$\begin{array}{c} d_{exp}=\frac{\lambda }{2sin\theta_{exp}} \end{array}$$

(3) $$\begin{array}{c} d_{0}=\frac{\lambda }{2sin\theta_{0}} \end{array}$$

The calculations give $d_{esp}=0.24$ and $d_{0}=0.23$, indicating that the incorporation of In led to an increase in the interplanar spacing, proving that In successfully doped the Ga_2_O_3_ lattice rather than existing as an impurity phase.

Based on the combined results of the peak intensity and grain size, the film with a thickness of about 105 nm possessed best crystallinity among the thermally annealed (GaIn)_2_O_3_ films. No evidence of phase separation was observed in this study. In contrast, (GaIn)_2_O_3_ films grown by MOVPE are prone to phase separation, as evidenced by the characteristic peaks of In_2_O_3_ in the XRD results. However, under specific conditions, In can be incorporated into the epitaxial layer, such as when H_2_O is used as the oxidant and the process is operated at relatively high reaction pressures [26]. This difference suggests that the non-equilibrium nature and high instantaneous deposition rate of PLD can effectively suppress In/Ga segregation during film growth.

**Figure 4:**
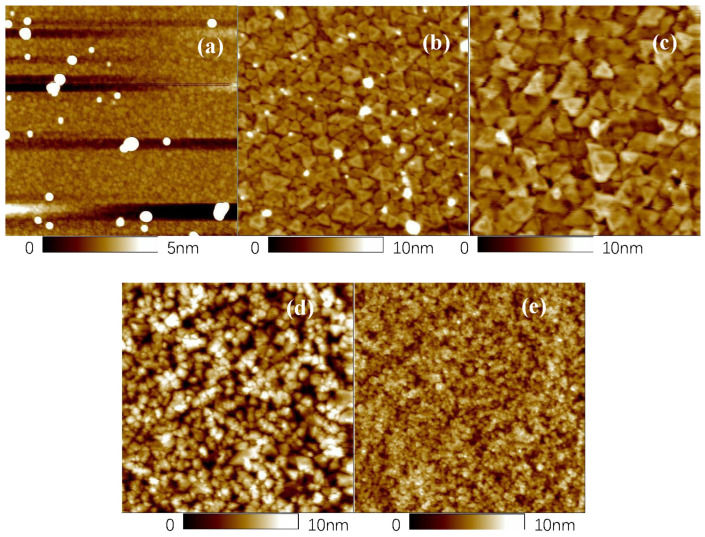
Surface images of (GaIn)_2_O_3_ films with thicknesses of (**a**) 20 nm, (**b**) 71 nm, (**c**) 105 nm, (**d**) 253 nm, and (**e**) 391 nm.

Figure 4 shows the AFM surface morphology images of the (GaIn)_2_O_3_ films with different thicknesses. The surfaces morphologies varied with the film thickness. The surface of the thinnest film (20 nm) was mainly composed small irregular grains, as shown in Figure 4a. With increasing the film thickness to 71 nm, the grain size became larger, and the grain shape turned to a regular triangle, as shown in Figure 4b. The triangle shaped grain size became even larger for the 105 nm thick film, as shown in Figure 4c. However, continuing to increase the film thickness decreased the grain size and randomized the grain shape, again as shown in Figure 4d,e. The variation in the surface morphology agrees well with that of the XRD results, indicating the variation in the crystallinity with the film thickness. The film with a thickness of about 105 nm showed the strongest XRD peak intensity as well as the largest grain size, indicating the best crystallinity out of the obtained films. Both decreasing and increasing the film thickness decreased the crystallinity of the (GaIn)_2_O_3_ films. The RMS surface roughness of the PLD-grown (GaIn)_2_O_3_ films exhibited a non-monotonic dependence on the thickness, with values of 2.040 nm, 1.270 nm, 1.043 nm, 1.921 nm, and 1.057 nm for thicknesses of 20 nm, 71 nm, 105 nm, 253 nm, and 391 nm, respectively. The lowest roughness was achieved at a thickness of ~105 nm, indicating an optimal balance between nucleation and grain coalescence in PLD growth. For comparison, CVD-grown films with a thickness of 37 nm have been reported to show an RMS roughness of ~1.03 nm [27], while sputtered films with a thickness of 200 nm exhibit an RMS roughness of ~1.088 nm [28]. These values are comparable to those obtained by PLD, suggesting that PLD can produce films with a surface smoothness on par with CVD and sputtering, despite differences in growth kinetics and deposition energetics. It is noticeable that a few bright protuberant parts can be observed in films with thicknesses less than 71 nm. The appearance of the protuberant parts is not clear at this stage, and this was temporarily attributed to the non-uniform elemental distribution during the early stage of PLD growth, where larger-diameter In atoms tend to deposit on the film surface.

**Figure 5:**
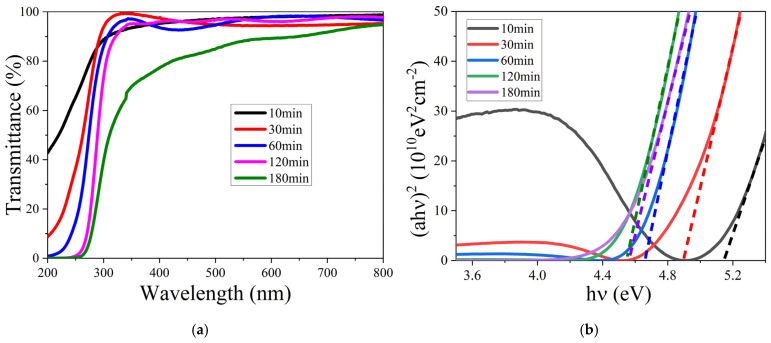
(**a**) Transmittance and (**b**) (αhν) ^2^ vs. hν of (GaIn)_2_O_3_ films with different thicknesses.

The transmittance spectra of the films with different thicknesses are shown in Figure 5a. It is clear that the absorption edge of the (GaIn)_2_O_3_ films shifts toward longer wavelengths with the increase in the thickness. Except for the film with a thickness of 391 nm, all the films show a high transmittance above 90% in the visible and infrared regions. The bandgap of the films was calculated by plotting (αhν)^2^ against hν, as presented in Figure 4b. The bandgap value of the films with thicknesses of 20, 71, 105, 253, and 391 nm are about 5.14, 4.91, 4.66, 4.56, and 4.55 eV, respectively. It is obvious that the bandgap decreases with the increase in the film thickness. The bandgap of the films at the initial growth stage is slightly higher than that of the reported (In_x_Ga_1−x_)_2_O_3_ films. As the film thickness increases, the growth mode transitions to a layer-by-layer growth, leading to an enhancement in film quality, with the bandgap converging towards the values reported for (In_x_Ga_1−x_)_2_O_3_ films. For example, the bandgap of (In_x_Ga_1−x_)_2_O_3_ (x = 0.035) grown by MOVPE is 4.52 eV. Despite the significantly lower indium content, this is still close to the lower limit of the results in this study [29]. The bandgap tuning range of (In_x_Ga_1−x_)_2_O_3_ films (x = 0~0.67) grown by Mist CVD is between 5.3 eV and 4.0 eV [30]. Furthermore, Liu et al. simulated (In_x_Ga_1−x_)_2_O_3_ films with x ranging from 0.0482 to 0.1875, and their bandgap tuning range was found to be between 4.817 eV and 4.422 eV [31].

From the above results, we found that the structural, morphological, and optical properties of the (GaIn)_2_O_3_ films varied with the film thickness. The variation in the structural and morphological properties indicates a change in crystallinity. For the films thinner than 105 nm, the crystallinity increased with the film thickness, as evidenced by the increase in the XRD peak intensity, as shown in Figure 3, and the grain size, as shown in Figure 4. This can be attributed to the large number of small nuclei formed during the initial stage of PLD deposition, which leads to a high grain boundary density and thus a lower overall crystallinity. The EDS results further reveal that the In content is relatively high at this stage; the substitution of Ga^3+^ by In^3+^ induces lattice expansion [32]. With further deposition, adjacent nuclei coalesce, reducing the total grain boundary area and enhancing crystallinity. At this stage, the In content in the film reaches a stable range, where moderate lattice expansion helps relieve interfacial stress and promotes grain growth [21]. Similar results have been reported by other groups. Kumar et al. attributed the poor crystallinity in thinner films to the incomplete growth of crystallites [33]. Yergaliuly et al. reported similar findings by depositing ZnO thin films. The film obtained after 50 deposition cycles exhibited the best overall performance. Films that were too thin suffered from incomplete structures, while excessively thick films showed increased defects and surface roughness [34]. However, for the (GaIn)_2_O_3_ films with thicknesses higher than 105 nm in this work, the film crystallinity deteriorated again. We suggest that with the further increase in the film thickness, internal stress accumulates due to factors such as differences in thermal expansion, defect proliferation, or lattice mismatch, leading to lattice distortion or microcrack formation. Consequently, the AFM observations reveal a reduction in grain size and an increase in grain boundaries. In addition, the deterioration in the crystallinity with the film thickness was attributed to the existence of oxygen vacancies in our as-deposited (GaIn)_2_O_3_ films. We have annealed (GaIn)_2_O_3_ films under different atmospheres. We found that the as-deposited (GaIn)_2_O_3_ films experienced a great oxygen deficiency, and the introduction of oxygen during the annealing process was helpful [16]. In this experiment, thicker films prevented the penetration of oxygen into the bottom side of the (GaIn)_2_O_3_ film, thus resulting in poor crystallinity.

The bandgap decreased with the thickness for the films thinner than 105 nm and remained stable for the films thicker than 253 nm. The variation in the bandgap could be due to the influence of various factors such as crystal structural, stress, carrier concentration, and deviation from stoichiometry of the film [35]. The shift in the optical bandgap for the (GaIn)_2_O_3_ films with thicknesses less than 105 nm in this work can be attributed to the effects of both grain size and stress. Chakrabarti et al. found that Bi_2_O_3_ powders show an increase in bandgap due to a reduction in grain size. The increase in the bandgap due to the reduction in grain size is due to quantum confinement [36]. Yildiz et al. revealed that grain size and strain significantly affect the bandgap of AZO nanostructures [25]. Viter et al. investigated grain-size-dependent bandgap shifts in SnO_2_ nanofibers. They suggested that quantum confinement and lattice strain in the SnO_2_ nanofibers were responsible for the bandgap shift [37]. In another aspect, the variation in the optical bandgap for the (GaIn)_2_O_3_ films thicker than 105 nm in this work should be related to lattice distortion and oxygen vacancy. The AFM measurements reveal that, with prolonged deposition, the (GaIn)_2_O_3_ films thicker than 105 nm developed lattice distortion and cracks, which increased the density of the grain boundaries and disordered states. Previous studies have found that Ga_2_O_3_ films exhibit a sharp change in bandgap during the transition from the amorphous to crystalline phase. X-ray photoelectron spectroscopy indicates that amorphous Ga_2_O_3_ films grown at lower substrate temperatures contain a higher concentration of oxygen vacancies and structural disorder. [38].

**Figure 6:**
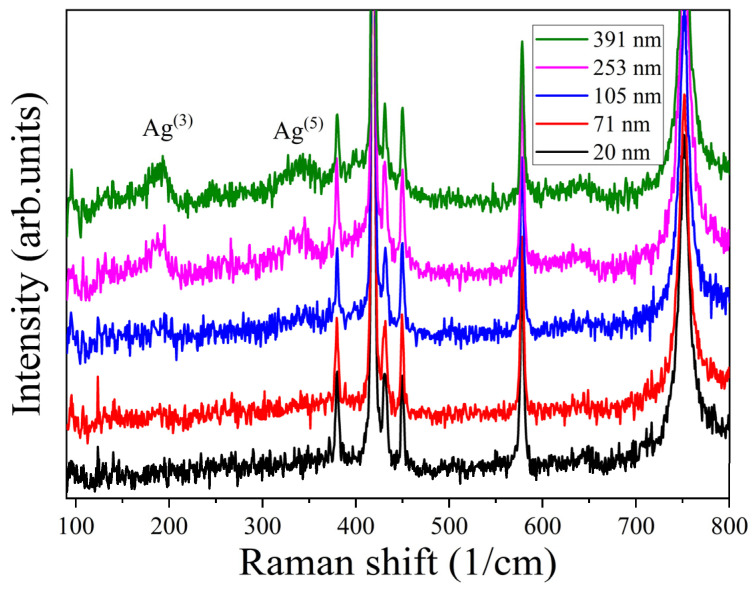
Raman spectra of (GaIn)_2_O_3_ films with different thicknesses.

The Raman spectra of the (GaIn)_2_O_3_ films with different thicknesses are presented in Figure 6. For the films thinner than 71 nm, no obvious Raman peaks belonging to (GaIn)_2_O_3_ can be observed. For the films with thicknesses higher than 253 nm, peaks located at 190 and 342 cm^−1^ can be observed, which can be attributed to the Ag^(3)^ and Ag^(5)^ phonon modes of β-(GaIn)_2_O_3_, respectively. The Raman spectrum can be influenced by many factors such as the crystallinity and film thickness. We have found that the Raman intensity of β-Ga_2_O_3_ increased with the increase in the film crystallinity [39] However, the crystallinity was not the main factor determining the Raman intensity in this experiment, because the film that possessed the best crystallinity (105 nm) did not show an obvious Raman peak. England et al. demonstrated that the intensity of Raman-scattered light follows a quantitative relationship with the thickness of Cr_2_O_3_ films [40]. The trend of our results agrees well with theirs, suggesting that Raman spectroscopy is a meaningful tool for estimating the film thickness.

## 4. Conclusions

(GaIn)_2_O_3_ is a multicomponent oxide semiconductor with a tunable bandgap that offers high optical transparency. The influence of thickness on film quality is multifaceted, being not only dependent on the growth mode of the deposition system but also on the competitive interactions between elements. However, to date, there have been no studies specifically investigating the influence of the thickness on the properties of (GaIn)_2_O_3_ films. This work investigated the effects of the film thickness on the morphology, strain, and defect states of (GaIn)_2_O_3_ thin films and elucidated how these structural variations contribute to the observed shifts in the optical bandgap. (GaIn)_2_O_3_ thin films deposited by PLD on sapphire substrates were annealed at 900 °C in air. The films thinner than 391 nm were of a high transmittance of over 90% in the visible region. The film with a thickness of about 105 nm showed largest grain size as well as the strongest XRD peak intensity, indicating the best crystallinity of that film. The bandgap value decreased from 5.14 to 4.55 eV with the increase in the film thickness from 20 to 391 nm. Both the crystallinity and bandgap value variation for the (GaIn)_2_O_3_ films thicker than 253 nm should be related to the oxygen vacancy. The results indicate that a thorough understanding of these thickness-dependent effects is crucial for optimizing device performance, offering valuable insights for selecting the optimal thickness that balances performance, surface morphology, and fabrication efficiency.

## Data Availability

The data that support the findings of this study are available from the corresponding author upon reasonable request.
